# “We Grind Each Other as Stones and Get Rid of Sharp Edges”: Young People’s Reported Positive Change, Learnings, and Growth Through Romantic Relationships

**DOI:** 10.5964/ejop.12735

**Published:** 2025-05-28

**Authors:** Nikola Kallová

**Affiliations:** 1 Institute for Research in Social Communication, Slovak Academy of Sciences, Bratislava, Slovakia; University of Palermo (UNIPA), Palermo, Italy

**Keywords:** romantic relationships, young people, learning, positive self-change, personal growth

## Abstract

Romantic and intimate experiences serve as a crucial learning ground for individual and relational development, yet many aspects of this learning process remain underexplored. The present qualitative study explores romantic relationship-induced learning among 104 participants between 18 and 35, which may foster better navigation of their future relational interactions. Data, i.e., 37 in-depth and 67 written interviews, were analysed using reflexive thematic analysis, applying an inductive, semantic, and experiential approach. The report of themes created in the analysis describes how romantic experience positively affected the participants in: (i) identity (self-knowledge, personal growth, and self-evaluation), (ii) relational competence (communication, socio-emotional skills, and problem-solving), and (iii) partner decisions (relationship expectations, relationship boundaries).

Previous or ongoing romantic and intimate relationships may contribute to the increased quality of subsequent close interactions (Tashiro & Frazier, 2003). Previous experience in a relationship can positively influence, for example, an individual’s communication skills, emotional management in passionate situations, the type of relationship they establish, and how they perceive and resolve conflicts (Shulman, 2003). Recognising the vital role of relationships in personal growth, development, and lifelong learning, there is a need for more robust scholarly debate—spanning transdisciplinary integrative qualitative research that expands, rather than further fractures, the existing theories and a comprehensive conceptual model to capture this phenomenon.

Research implications arising from existing studies on positive self-change from intimate relationships call for such an intertheoretical model that covers not only the results of relationship termination (e.g., Blackie & McLean, 2022; Tashiro & Frazier, 2003) but also the results of relationship types and changes. To address the criticism, we contribute to the developing discourse with a study based on the participants’ subjectivity, considering it a reliable and credible source of information (Gough & Madill, 2012) and a comprehensive, holistic, and in-depth investigation encouraged by Jamison and Sanner (2021). In doing so, we anticipate the unification of partial findings and pave the way for a further quantitative inquiry into learning from romantic relationships based on a conceptual model of this specific phenomenon instead of co-created measurement tools.

## Relationship-Induced Learning: Theoretical Perspectives

The assumption of the influence of romantic and intimate relationships on individuals’ quality of life and relationships began to be postulated and empirically tested in the 1980s and 1990s (Furman & Simon, 1999; Furman & Wehner, 1997; Johnson & Bradbury, 2015; Sroufe & Fleeson, 1986). The research literature generally conceptualises this phenomenon as: (i) learning or lesson-learning, (ii) self-development and personal development, or (iii) personal growth. This research article uses ‘romantic relationship-induced learning’ (RRIL) as a topic-driven umbrella term to encompass distinct concepts studied independently across psychological subdisciplines, aiming to facilitate cumulative research and integrative analysis without reinforcing existing theoretical fragmentation. RRIL refers to any relationship-induced improvement in personal skills, perspectives, experiences, or the acquisition of positive attributes, including building competence, pursuing and achieving specific goals, or developing new aspects of self-concept (Jakubiak & Tomlinson, 2020).

Although research on the positive consequences of romantic and intimate relationships is at the peak of a second wave of scholarly interest, partly due to the discourse on personal growth, the results are partial and mixed (see Jiang et al., 2022). Research on personal growth as a result of intimate relationships has been limited mainly to posttraumatic, ambivalent, or challenging events associated with breakups (e.g., Blackie & McLean, 2022; Tashiro & Frazier, 2003). The social/cognitive learning theories (Bandura, 1977, 1978) that have dominated this issue, particularly in the past decade, do not distinguish the full range of acquired knowledge or skills. Furthermore, they are currently limited primarily to explaining negative partner phenomena such as violence and aggression (Dim & Elabor-Idemudia, 2021), online abuse (Van Ouytsel et al., 2020), or criminal behaviour in intimate and romantic relationships (Giordano, 2020). The developmental literature, in turn, is limited to development related to personal identity (Aron et al., 1995; Furman & Shaffer, 2003) and selected personality changes (Neyer & Lehnart, 2007; Szwedo et al., 2022). Among the scholarship on the topic so far, a particular concept worthy of mention is the so-called Michelangelo phenomenon (Bühler et al., 2020; Rusbult et al., 2009). Closely related to both social learning and personal growth, it seeks to explain the process of how people in romantic relations adapt to one another and promote or inhibit each person’s pursuit of an ideal self; during the course of interaction, changing their behaviour as to coordinate with one another and respond to each person’s needs and expectations (Rusbult et al., 2009).

From a developmental perspective, the experience of romantic and intimate relationships is thought to influence personal identity, self-concept, self-esteem, self-image, or self-confidence (Erikson, 1968; Furman & Shaffer, 2003). Aron et al. (1995), in their prospective longitudinal study of self-concept change, found that falling in love is followed by an expanded self-concept and a change in self-concept evaluation that includes greater perceived self-efficacy and self-esteem. Other sources suggest that the identity development process due to intimate relationships may involve adopting moral and religious values, political ideology, career orientation, or changes in sexuality (Furman & Shaffer, 2003). The impact of relationships on personality development has also been addressed by Neyer and Lehnart (2007). They found that the transition into a first serious relationship moderates personality maturation, such as a decrease in neuroticism and shyness or an increase in extraversion and self-esteem. Relationship events have also been found to influence Big 5 personality traits (Asselmann & Specht, 2020).

RRIL also involves the development of relational competence and relational decision-making. For example, dating and relationship experiences in adolescence predict the quality of partner interactions in young adulthood (Szwedo et al., 2022), such as negotiating conflicts to mutual satisfaction or effective and timely caregiving (Madsen & Collins, 2011). Other qualitative research on personal growth has found that positive post-breakup changes, that may improve future relationships include changes in the individual (i.e., self-confidence or independence) in addition to relational changes (i.e., relational wisdom and skills, or communication skills), and changes in partner criteria, expectations, or caution in choosing a partner (Tashiro & Frazier, 2003).

## Method

### Study Design

The study intends to qualitatively document romantic relationship-induced learning in individuals that may improve future romantic encounters. Of interest are changes in individuals’ attitudes and behaviours that may cause higher-quality subsequent romantic experiences with the same or the next partner. Romantic experience is broadly understood as any subjectively reflected significant experience with a person of romantic interest or involvement. It can be a pleasurable experience with a long-term partner, a romantic break-up or rejection, or challenging events in a short-term affair.

### Recruitment Process and Data Collection Procedures

We targeted individuals with high priority for romantic relationships in key developmental periods for relational development. To be included in the sample, participants had to meet two criteria—being between eighteen and thirty-five years of age (i.e., the age group of emerging adulthood and/or early adulthood) and having an experience of a romantic relationship maintained for at least half a year. This specific age range aligns with the developmental stage, where individuals experience significant changes and transitions in various life domains, characterised by exploration, identity development, and establishing intimate relationships (Arnett, 2014; Erikson, 1963, 1968; Konstam, 2007; Shulman, 2017). By including participants who have had a romantic relationship lasting at least six months, we aimed to capture individuals with substantial experience in committed romantic partnerships, assuming differential self-change following varying relationship experiences (from less to more committed).

Two independent recruiters were contracted to recruit participants, and, following the given instructions, 30 individuals who met the age requirement, had been in a romantic relationship for at least six months, and had equal representation from eastern, central, and western Slovakia were selected. The remaining 74 participants were recruited through a snowball method, where participants were asked to target a diverse range of acquaintances who fit the study criteria. This approach targeted a varied sample regarding gender, student status, and geographic location within Slovakia. Data collection involved personal interviews with 37 respondents and self-moderated written interviews with 67 respondents. The decision to collect written data was motivated by participants’ preferences for privacy, reflection time, and the COVID-19 pandemic restrictions that prevented further face-to-face interviews (as the data collection took place in late 2019/early 2020). By offering multiple data collection methods, we allowed participants to choose the format that best suited their comfort and availability.

A co-created relationship history timeline was employed to guide the interviews and document relationship histories, similar to the approach used by Jamison and Sanner (2021). This graphical representation aided the reporting by providing a visual aid for participants to recall and detail the timing and duration of their romantic and sexual involvements. The interviews consisted of an introductory narrative and a semi-structured section with open-ended questions. The introductory narrative part encouraged participants to reflect on the most significant turning points and the role of intimate or close relationships in their personal stories. The semi-structured section covered various aspects of relationship history, such as understanding intimacy and relationships, important milestones, and experiences in current relationships and future planning.

Several questions were designed to directly ask about romantic relationship-induced learning, i.e., 1. *What did you take away from your significant relationships?* / 2. *Do you have any regrets about your past relationships, or is there anything you wish you had done differently?* (For the full version of the interview scenario, see Kallová, 2023). The interview duration ranged from 60 to 150 minutes. The questions of the self-moderated written interview mirrored the topics in the face-to-face interview. The face-to-face interviews were recorded and then transcribed. Data were then coded and analysed using Atlas-ti software.

### Participants

The final number of 104 Slovak-speaking participants comprised sixty-three women (making up 60.4% of the total) and forty-one men (39.6%) with an average age of 25.24 years. For more demographic sample characteristics, see Table 1.

**Table 1:** Demographic Sample Characteristics and Percentages

Characteristic	Percentage
Religion	Religious (78.4%)
	Non-religious (21.6%)
Occupation/status	Students (41.7%)
	Working (49.52%)
	Students also working (3.9%)
	Unemployed (4.9%)
Residence	Rural areas (49.5%)
	Urban areas (40.8%)
	Large cities (9.6%)
Education	Primary education (5%)
	Secondary education (45%)
	University education (50%)
Relationship status	Unmarried relationship (63.5%)
	Single (23%)
	Married (13.5%)
Sexual orientation	Heterosexual (96.2%)
	Same-sex orientation (3.8%)

When attempting to understand the experiences of romantic relationships in Slovakia, a brief mention of the sample’s cultural, religious, and sociopolitical context is in place. Conservative values, the influence of Catholicism, and challenges faced by LGBTQ+ individuals may shape relationship expectations and the ability to express one’s identity in romantic relationships openly. The traditionally inclined context and the current political climate favour beliefs about prescribed and predefined gender roles. RRIL builds on the widespread cultural models and promotes normative structures embedded in the “right” partner behaviours.

### Data Analysis

Data were analysed following a six-step reflexive thematic analysis (RTA) procedure (Braun & Clarke, 2006, 2019, 2022). Reflexive TA is a flexible method for identifying, analysing, and creating patterns (themes) within data (Braun & Clarke, 2006) and is widely credited for allowing reflexivity, subjectivity, and in-depth study. Reflexive TA is often used in qualitative research in psychology, health, and other disciplines (Braun & Clarke, 2014). We viewed the data through the prism of essentialist ontology and critical-realist epistemology. As for the analysis pattern for the essentialist approach, Majumdar (2022) summarises that it should be more subjective in nature as its focus is more straightforwardly placed on individuals’ interests, motivation, meaning, and experiences. In the dataset’s analysis, credibility and validity were thus attributed to participants’ subjective reports of their account of reality and how they made sense of it. Experiential orientations to data have been adopted; therefore, we rely on participants’ subjective statements to indicate romantic relationship-induced learning. As a result, primarily inductive and semantic coding has been employed to study the phenomena. Individual analytic steps are described in Table 2.

**Table 2:** Phases of the Reflexive Thematic Analysis

Phase	Interview Analysis Procedure	Examples
1. Familiarising with the data	The team of 8 researchers familiarised themselves with the data set through re-readings, note-taking, and collective discussions to better understand the data, its patterns and meanings, and how others view them.	A phenomenon identified at this stage as worthy of further attention was the educational potential of relationships.
2. Data coding	The research team collectively created a preliminary code book that mapped, organised, and grouped different areas of interest in the texts. The coding process was conducted inductively using open and in vivo coding techniques, labelling relevant and distinct text passages. The research pairs reviewed and discussed their assigned codes following the general coding.	Over 200 preliminary codes were created for the study (e.g., “intimate partner relationship transition”), reduced with each step by merging and discarding, and renamed with evolving conceptual clarity (e.g., “identity changes,” “partner skills enhancement,” “relationship decisions,” etc.).
3. Theme creation	The grouping of codes that shared common patterns and meanings led to the creation of the first themes. Revisiting and revising codes allowed some to be grouped into sub-themes, others to form separate themes and others to be merged into a single sub-theme or removed from further consideration.	Themes like „Wanted and unwanted partner behaviours” or “Identity or Who am I” were created.
4. Theme revision	The draft themes were further revised by the research pair, with some being transformed into subthemes, others into separate themes, and some merged or removed to ensure internal homogeneity and external heterogeneity.	Themes were revised, regrouping codes and preliminary themes based on the type of romantic relationship-induced learning.
5. Defining and naming themes	The manuscript author defined and named the themes after carefully considering each theme's content and characteristics to capture the essence of the data accurately.	Descriptive working theme titles (e.g., “Desired and undesired partner characteristics/behaviours”) were changed into interpretive titles (e.g., “What I want and don't want in a partner”).
6. Writing of the report	The manuscript author was involved in writing the report, which focused on presenting arguments and findings related to the research questions.	Answering the question “What types of lesson-learning and gaining insight following a relationship encounter have the participants reported?” was the purpose of the final analysis stage.

### Ethics

The Ethics Committee of the Institute for Research in Social Communication approved our study. All participants provided written informed consent prior to enrolment in the study. Participation in the research was voluntary and confidential. Before the start of the research, all participants were acquainted with the purpose of the study and how the data would be handled. All procedures carried out in the research involving human participants were in accordance with the 1964 Declaration of Helsinki and its later amendments or comparable ethical standards.

## Results

Based on qualitative data analysis, the study documents romantic relationship-induced learning reported by participants that may enhance future romantic encounters. Based on their evaluations of their romantic experiences, the participants perceived these changes as formative, beneficial, and enlightening.

“I am grateful that I learned from her how the relationship should look, what the person should feel, how he should behave, how not to behave. I’m grateful for that. I’m probably the most grateful for what I’ve been through with a girl. I know what I felt, and I know I don’t want to feel anything less. It may be naive, but that’s how I’m set up.” (D15 M27)

Consistent with the literature on adversarial growth (Blackie & McLean, 2022; Tashiro & Frazier, 2003), however, the vast majority of instructive situations for the participants occurred in unpleasant relational events such as infidelity, break-up, or conflict. A number of participant quotes very aptly capture and summarise the saying retold by the author of the following one: what doesn’t kill you makes you stronger, which implies, among others, increased resilience as a resulting change.

“... when they cheated on me, I was suffering ... How good, isn’t it? What doesn’t kill you will definitely make you stronger. (D6 M32) / I don’t know, there's nothing to regret. Probably not. We learned something. It was a long, winding road. Such a detour, but we learned something. (D20 F29) / And basically, I have a positive view, or rather a learning-positive view, of virtually all of them and basically of all of those women and relationships. I just wouldn’t say these were happy relationships.” (D4 M27)

In the analysis, three themes were developed to denote romantic relationship-induced learnings described by the research participants that may benefit them in future relationships. These are:

a) “Who I am and where I’m headed” (including sub-themes: self-knowledge; personal growth; and self-evaluation).

b) “The kind of partner I want to be” (including sub-themes: communication; socio-emotional skills; and problem-solving).

c) “What I want and don’t want in a partner” (including sub-themes: relationship expectations and relationship boundaries).

### Who I Am and Where I'm Headed

“So it gave me that. Knowing what I don’t want to be and what I want to be. I realised that pretty late. Those relationships hadn’t been very serious before, so I hadn’t even had time to study myself in depth.” (D8 F26)

The opening participant’s quote is noteworthy in the suggestion that an in-depth “study” of the self is tied to serious romantic relationships as a fruitful backdrop for framing self-understanding. According to the participants, their romantic relationships had a significant impact on them in terms of their identity. Chryssochoou (2003) defines identity as a relationship between the individual and the world, which has three components: “It includes an element of cognition (self-knowledge), answering the question ‘What do I know about me?’, An element of self-action pertaining to the claims I want to / can make about myself and an element of Other(s) actions that recognize me and allow me to make the claims I wish to make about myself and to be who I want / think myself to be” (p. 228). Statements reflecting all three elements of identity—ranging from introspective to socially oriented—were present in the sample. Through their experiences in romantic relationships, participants developed ideas about who they are, what they aspire to or wish to avoid becoming, and how they perceive themselves in light of how others relate to them.

“It was a nice time during which I somehow formed out.” (D55 F24)

#### Self-Knowledge

As for the element of cognition, many participants report that they got to know themselves better through romantic relationships. “Who am I? What am I like and what not? What do I want, like, or do not like?” They express that their answers to these questions and this type of self-knowledge are influenced by and/or originate in current and past partnerships or the separation process.

“It simply came to my notice then what I am like. Most of all, I recognised my shortcomings, so we can consider it self-knowledge, but I also recognised my strengths, not just the bad things. My first self-knowledge began mostly in the fifteenth year of my life when I had a relationship that might have had some perspective, but since that didn't happen, I at least got to know my weaknesses in my social side, which I should work on.” (D86 F21)

#### Personal Growth

The statement belongs to a participant who bridges from the cognitive element of identity to the element of the act (self-action). Specifically, it is an act of desired change, which aligns with the definition of personal growth in several areas. Growth within the self that participants describe ranges from changes related to the intellectual faculty, mental state, or sexuality to other types of abilities and skills in the social or artistic sphere to character strengths.

“A very crucial step in shaping my personality was also my love, thanks to which I got rid of certain addictions and changed the bad habits and inappropriate ways I had, and made me a better person.” (D110 M20)

Relationships have also helped several individuals to grow in autonomy, facilitating such shifts as they transition to adulthood or independence from their parents.

“Thanks to my partner relationship … I started to follow my own reason, my own intuition, to rely more on myself. Be more responsible. I disengaged under the influence of my parents. Now they respect my decisions.” (D85 F22) / “I feel that perhaps through this relationship and by spending a lot of time together, I matured much earlier than some of my classmates.” (D55 F24)

There are participants' statements in which the growth they describe took place spontaneously without reflecting on the need that would precede it or a deliberate effort for a change. Others, however, mention the need to change or improve themselves. Self-reflection and an awareness of the changes one wants or plans to make or should undergo occurred across the sample. One of the participants says this is given by what he calls ‘feedback on life’ that stems from the relationship experience. Another one elaborates on the same phenomenon as follows.

“In this relationship, lasting five years, I was told everything by my ex-boyfriend. That was how I functioned, what I was like ... and he told me terribly the things I realised in retrospect that it was true, that's all wrong. That’s exactly who I am. And it's all me saying, ‘Yes, I’m such that I can change something and something not.’ And in intimate things, he also told me that you are so and this and that .... And then I started to realise it ... That it is such that, Jesus, that it is right ... and always I think to myself, ‘[name], change, you can’t be like that, because it's probably bad.” (D12 F28)

#### Self-Evaluation

Identity influenced by partnerships also includes the third of the aforementioned elements of identity, namely attitudes toward oneself and self-evaluation based on other people’s approaches. Participants talk at length about the actions of their partners that contributed to the construction of self-confidence, self-esteem, self-respect, and self-value. In our sample, the impact of romantic relationships on self-perception was characteristic of women. The reevaluation of their self has, in turn, led to a change in how they would want to be treated and their assertiveness.

“... maybe out of self-esteem. I already know that I can say something. That I’m not 20 anymore, and I won’t be quiet. Ok, so you like it, so I’ll be quiet? That I’ll say something. That is the change. That I already know what I like. I didn’t know that before.” (D20 F29)

### The Kind of Partner I Want To Be

Unlike the first theme, the second theme refers to knowledge and skills acquired during romantic encounters that are applicable directly and solely in subsequent relationship events. The first broad and vital area of relational competence gained from the romantic experience is communication with a partner.

#### Communication

Through a relationship experience, participants can improve their communication skills or develop new ideas about communicating with their partner, which they can later apply to their relationship to improve its quality. The present research identified a large number of statements on such self-change and RRIL. Participants became particularly aware of the need to listen to their partner, improved their ability to listen to their counterpart, re-evaluated the importance of communication, openness, and verbalising disagreement for the relationship, and practised self-expression.

“It wasn't until we broke up that I realised that it was necessary to talk to each other well enough. This relationship has taught me to express my feelings, to express when I don't like or mind something, to deal with it together and not to put it off, not to bother with things only in my head.” (D89 F22)

#### Socio-Emotional Skills and Problem Solving

Based on participants’ reports, in relationships and breakups, young people may become aware of / instructed and/or trained in: (a) their emotional regulation or (b) prosocial orientation and behaviour. These socio-emotional abilities include but are not limited to honesty, problem-solving, and striving for both a moderate level of adaptability as well as continuous maintenance of the quality of their partnerships. The following participant's quote demonstrates his awareness of the need to work on the relationship but also includes a description of the stages of emotional regulation, a problem-solving orientation, and a mention of effective coping strategies, all of which he learned from his relationship experience.

“Not to give up, like if it's very bad now, but try to handle it internally, or bridging it into some kind of opportunity in that relationship, or especially elevating those of your mental dogmas, like ... Ah, so this is a situation where I am angry, and now I'm going to try to do it differently. I'll try to take it, um, not how I would take it at first, but rather think about taking a break from that discussion, going back to it, thinking about it from a different perspective.” (D16 M31)

In contrast, another participant indicated that the relationship made her realise how she does not want to behave in a partnership. The relationship proved to be a fruitful venue for her to gain different attitudes towards the abusive partner behaviours she manifested. She became keen to correct her behaviours, such as manipulating others to attract their attention by lying, authoritarian behaviours, or exploitation.

“I took a lot of things from it as far as my behaviour towards people is concerned. I never want to behave the way I did to him again. I admit that I am not proud of myself for some things. I have even been making something up, for example, to get his attention. I don't want to do that in my life anymore. I try to work on a lot of things, such as how I treat people or how I use the goodness of my partners over time … sometimes I try to get the most out of it, and it’s disgusting, terribly disgusting. Then as if I expect them to always be good to me and to benefit from it.” (D8 F26)

The same participant goes on to express that the problems in her relationships were due to her behaviour and that if her future relationships are to improve, changes must occur in herself first and foremost.

“I really have so many things in mind now when I meet someone new ... I have to change so that it doesn’t turn out the same way ... It gave me a lot of self-reflection, such as to think, to realise things I have to change when I want to have a normal partner in life. For life. Who would stand it with me without any harm to the psyche. So it gave me various things that bother me, but I take it positively. So what, I made mistakes, and I’m glad I’m not in that relationship anymore and that I’m not going to make them in the next one.” (D8 F26)

### What I Want and Don't Want in a Partner

Participants’ accounts helped to understand the range and role of decisions and choices one makes about choosing a partner and maintaining a relationship. Firstly, an entry into new partnerships can be (consciously) influenced by previous relationship encounters. After a previous experience, most frequently an unpleasant one, many participants promise prudence, patience, and vigilance when choosing another partner. The data show that the personal decision whether or not to remain single depends, among other things, on previous relationships. The same applies to how the participants evaluate romantic relationships’ importance for flourishing.

“After this last relationship, I changed my whole view, I realised that the relationship is not so important for me because I’m mostly unhappy in them, so if I really go into a relationship, I want to be absolutely sure that it has perspective and it’s really serious.” (D73 F22)

#### Relationship Expectations

In addition to whether and under what circumstances it is adequate to enter into a relationship, previous relational experiences bring about a new set of partner expectations, serving as a guide for new and future relationships.

“Each new relationship began with a new experience, and I could be alert to the experiences I had already had.” (D46 F21)

In both the relationships that are primarily constructive and healthy for the partners, and in those holding pathological features, individuals form a picture of what their (next) partner should and should not be like and how they should or should not resemble a previous or current partner. This includes the partner behaviour they exhibit and the personality of a partner suitable for the courtship. Some essential criteria are partner character traits, age, place of residence, or the nature of the emotion and bond they provide. Among the valued requirements are partners’ unconditional love towards them or partners’ willingness to be helpful, and among the characteristics they plan to avoid in the future are remote partners providing long-distance relationships and partners who tend to humiliate or manipulate the other in a pair.

“And once I had one Tinder date ... And I don’t even know how to describe it, but it was like, wow, it might look like this, as if my expectations of how nice it can be are growing because then one no longer wants to go under those expectations.” (D20 F29)

#### Relationship Boundaries

As in the previous participant’s case, a romantic experience affects the construction of expectations or the formation of partner ideals. The same goes for setting rules and limits, or ‘what belongs to a relationship and what does not,’ as the author of the following statement puts it.

“I take a lot from the first relationship, I think, because that was the moment when I learned how to ‘live’ in a couple, what belongs to a relationship and what doesn’t, and I entered the second relationship with the fact that I already knew what and how. What can hinder the other in the pair, and what can be expected from a man. My boyfriend calls it very well that we grind each other as stones and get rid of those sharp edges of habits, things that may bother the other.” (D55 F24)

This phenomenon appears in the data either as a constant ‘calibration’ of partners’ behaviour in the relationship, which was the case in the quote above or as a setting the boundaries in the individual that guide them in deciding which partner attributes and behaviours they accept and which they no longer accept.

“Every relationship has taught me something, pushed me further, taught me what can be taken more seriously and what can be ignored in a light-hearted way.” (D49 M31)

## Discussion

This qualitative study aimed to document participants’ romantic relationship-induced learning—positive self-changes resulting from their romantic experiences—which may serve as antecedents to enhanced quality in future relationship encounters. We found that a romantic experience was formative for almost all participants. The report of themes created in the analysis describes how romantic experience affected the participants in the following.

1. Identity, such as self-knowledge, personal growth, and self-evaluation (**Theme A**: Who I am and where I'm headed) which has been scientifically proven to be beneficial for the quality of romantic relationships (Farooqi, 2014; Furman & Shaffer, 2003).

2. Relational competence, such as communication, socio-emotional skills, and problem-solving (**Theme B**: The kind of partner I want to be) which, according to previous research, significantly affects the relational quality (Batool & Khalid, 2012; Farooqi, 2014; Frei & Shaver, 2002).

3. Partner decisions (partner expectations and partner boundaries) (**Theme C**: What I want and don't want in a partner), previously found to be determinants of the quality of a romantic relationship either in the form of potential partners’ psychological conditions, character strengths, childhood, and family background, or their attachment style (Boiman-Meshita & Littman-Ovadia, 2022; Egeci & Gencoz, 2011; Feeney & Noller, 1990; Fitzpatrick & Sollie, 1999; Xia et al., 2018).

Regarding the first theme, findings support and complement previous studies on relationship experience affecting an individual’s identity, personal growth, and different self-concept areas (such as self-esteem, self-confidence, self-respect, and self-value) (Aron et al., 1995; Furman & Shaffer, 2003; Slotter et al., 2010; Tashiro & Frazier, 2003). Our findings also support other researchers’ views on independence as a possible consequence of romantic relationships (Tashiro & Frazier, 2003). Unlike most previous studies, however, our findings were not limited to adversarial growth and self-changes from the negative partner experience. We found that self-knowledge, independence, maturation, and personal growth generally can be fostered in well-functioning relationships as well as in problematic ones (Lee et al., 2018).

Jamison and Sanner (2021) state that nowhere is identity development and self-understanding more salient than in the context of romantic relationships, which require that individuals identify what kind of partners they want, what kind of partners they want to be, and how to maintain individuality in the context of a relationship. On a related note, Norona et al. (2017) state that past romantic experiences can teach individuals about what they do and do not want in the future. Similarly to these claims, we titled the second theme “The Kind of Partner I Want to Be”. Relational competence, a subject of the second theme, has been previously studied as an outcome of romantic encounters (Madsen & Collins, 2011; Shulman, 2003; Szwedo et al., 2022; Tashiro & Frazier, 2003). Our findings align with Norona et al. (2017) study that describes relational competencies such as compromising, give and take, working on the relationship, and awareness of each couple member’s needs as practical relational lessons for romantic relationships as they require active work from both partners to fulfil each other’s needs. Norona et al. (2017) further mention communication in its nuanced forms, such as openness to self-disclosure or allowing romantic partners to share their opinions, and also the dual importance of being honest with romantic partners and trusting that they will still be accepting, as well as reciprocating that trust and acceptance. Our study mirrors these findings and found communication, which includes verbalising disagreement, listening ability, and honesty, as a vital relational competence learned by our participants in their relationships. Furthermore, we found other socio-emotional skills such as emotional regulation, prosocial orientation and behaviour, and problem-solving mentioned by the participants as other types of relational competence and interaction that are subject to change due to RRIL.

The third and last theme also deepens and widens previous findings in that our results not only support the thesis on relationship break-up affecting future partner criteria, expectations, or cautiousness in choosing a partner (Tashiro & Frazier, 2003) but also the experience of a well-functioning relationship may contribute to forming a picture of what the desired relationship or a partner should be like. In their analysis, Norona et al. (2017) found that individuals’ break-up experiences resulted in their decision to trust with caution, in a balanced manner, at an appropriate time in the relationship, and remain vigilant early on in relationships. Their interpretation is that this lesson reflects regulatory processes that one might take to pursue relationships more slowly. We found that similar regulatory processes occur due to positive relational experiences in our participants’ cases. Not only break-ups and arguments but also other types of romantic experiences shaped their relationship expectations and helped them define their boundaries in future partnerships. They gained clarity about their desires and deal-breakers, enabling them to make more informed decisions when choosing a partner. These findings touch on how previous relationships may influence opinions and decisions on singlehood and the role of romantic relationships in their flourishing.

Partner decision-making and the changes in identity and partner competence induced by relational experiences have their place in professional and practitioner discourse. Our approach unified distinct concepts—typically studied independently and partially across psychological subdisciplines—under the umbrella term of romantic relationship-induced learning, grounding it in participants’ natural language and perceptions. Such an approach is advantageous for understanding how the different concepts and components of romantic and intimate relationships interact and perform related functions (Jamison & Sanner, 2021).

### Limitations

Self-reported data and the retrospective design allowed for the holistic identification of perceived romantic relationship-induced learning. On the other hand, as a limitation of the study, this type of exploratory research does not allow for the observation of actual relational learnings, which are subject to academic critique (Owenz & Fowers, 2019). Additionally, smaller sample sizes, characteristic of in-depth qualitative studies, limit the ability to draw conclusions based on quantity-driven data, such as comparisons across demographic characteristics. Further research with a larger, representative sample would be necessary to fully explore potential demographic and cultural differences related to the phenomenon.

A significant group of participants engaging in a written rather than a face-to-face interview can be seen as a further limitation. Whilst this approach facilitated a sense of privacy and a safe space for participants to share intimate details about their lives without being confronted by the presence of an interviewer, participants might have been consequently motivated to be less emotionally engaged and dense in terms of the breadth of their accounts. Moreover, no probing questions could be asked by the interviewer.

### Conclusions

The present study’s findings complement previous research on the influence of relationship experiences on individuals’ identity, relational competence and relationship decisions and expand upon existing knowledge in several areas. Unlike previous studies that primarily focused on adversarial growth and RRIL from negative partner experiences, we found that positive changes and growth can occur in both well-functioning relationships and problematic ones. This suggests that individuals can experience changes in identity (self-knowledge, independence, personal growth, expended self-concept or self-evaluation), relational competence (communication skills, such as verbalising disagreement, active listening, and honesty, socio-emotional skills like emotional regulation, prosocial orientation, and behaviour, as well as problem-solving abilities) and form their partner expectations and boundaries in a variety of relationship contexts.

The findings of this study have several implications for future research on romantic relationships. By bringing together concepts that have been studied separately across psychological subdisciplines, the study promotes a more integrated understanding of relationship-induced learning. It also highlights the importance of considering both positive and negative experiences in relationships when examining their impact on individuals. Ultimately, the study provides a clear conceptual foundation for future endeavours to define and measure the dimensions of constructs falling under the umbrella term ‘romantic relationship-induced learning’.

Research implications call for testing relationship-induced learning due to different relationship types and changes. The rate and learning from intimate relationships could underlie different types of ongoing relationships besides romantic breakups, such as noncommittal dating or casual sex versus long-term relationships, the impact of which has not yet been confirmed (Beckmeyer & Jamison, 2021; Jamison & Sanner, 2021). The authors bring awareness to sexual identity, development, and exploration, as an integral part of relationship development. That leads them to the idea that casual relationships and hookups may be particularly important in helping individuals develop a rubric for selecting compatible partners and understanding what individuals need and want from relationships.

Criticisms of existing research on this topic also focus on the almost exclusively retrospective design of the studies conducted, and there is a need for a longitudinal research approach to this topic (Owenz & Fowers, 2019). Individuals’ success in rectifying problems from one relationship to another may depend on their attributions regarding why the previous relationship ended (Tashiro & Frazier, 2003). This lends credence to perceived and reported changes and their narrative production as an area for more future studies on this phenomenon.

## Supplementary Materials

For this article, the following Supplementary Materials are available:

- Study materials. (Kallová, 2023)

## Data Availability

The source data is stored with the project leader. Only members of the research team have access to a copy of the full interviews for the purpose of analysis, subject to written consent from the participants. The participants have not provided consent to publicise their interviews in their entirety. Participants gave consent to use anonymised data excerpts for the purpose of publication. Material containing information on data collection are stored at: (Kallová, 2023).
